# Neuroanatomy of the Will

**DOI:** 10.3390/neurosci3040044

**Published:** 2022-11-07

**Authors:** James William Hendry Sonne

**Affiliations:** Greenville, SC, USA; jamessonne@gmail.com

## Abstract

Questions regarding the nature and source of consciousness and individual agency to make decisions have enormous practical implications that include human health and wellbeing, social policy, and economics. Ethical issues involving the ability for patients to make conscious, informed choices, such as in cases of dementia or coma, abound, and the health implications of individual choice on public wellbeing are becoming increasingly important as population densities increase. Furthermore, the use of animals for drug testing presents moral dilemmas related to our concepts of consciousness, pain, and consent. While philosophers have long debated aspects of consciousness, the means to scientifically address specific questions regarding regional and cellular functions of the brain are constantly emerging, as are new theories of physical laws and particle interactions which allow for the formation of new hypotheses of the source of consciousness. These emerging capabilities and hypotheses are increasingly able to be subjected to methodological scrutiny by the scientific community. To facilitate open discussion and advances in investigations regarding the nature of consciousness, this Topical Collection is intended to provide a peer-reviewed space to discuss or propose falsifiable hypotheses of consciousness in a full range of systems, using methods across disciplines of biology, physics, computer science, and philosophy of science that can inform such a discussion, while emphasizing the role that our conception of consciousness has on human health, society, and policy.

## 1. Introduction

Consent and individual agency regarding the ability to make a free choice are paramount to numerous aspects of modern life, ranging from health and medical treatment to legislative policy and justice. However, these critical aspects of human consciousness are often assumed to exist, or not to exist, and are poorly defined with no agreed-upon ability to validate their existence. Some of the most devastating and impactful health maladies are those that impact a patient’s cognitive and mental abilities, personality traits, and memory, such as Alzheimer’s disease, depression, post-traumatic stress disorder, anxiety, schizophrenia, coma, and many others. When a patient’s memory, executive function, or motivation for treatment are impacted, the ability of modern medicine to treat, and of biomedical research to understand, these vulnerable patients who are unable to provide individual consent is impacted [1]. The ability of researchers and clinicians to present treatment efficacy, and ultimately realize the advancement of medical science, requires that patients consent to treatment and the sharing of their medical data. When unable to do so individually, institutions resort to consent by proxy, such as family members of the patient or potentially by institutional review boards. This concept has extended to institutional committees providing proxy consent for animal research with the intention of advancing biomedical science [2].

Extending beyond individual health, individual choice is sometimes at odds with pro-social actions related to public health initiatives. Childhood vaccinations are nearly universally accepted in the United States [3], but there exist small or isolated population groups that choose to refuse vaccines [4]. From a public health perspective, vaccine refusal may be considered irrational due to the high efficacy and low rates of adverse effects observed in clinical trials, but from the perspective of the individual, the refusal of a vaccine for an otherwise rare disease may be logical based on some information or individual situation. A better understanding of individual agency and the factors considered when individuals make choices for themselves that impact public health may influence our understanding of individual concerns. This knowledge can potentially lead to changes in the myriad aspects of public health initiatives to improve the individual uptake of treatments such as vaccines. Perhaps methods of administration or drug formulations could be varied to increase acceptance among groups in society, or public health campaigns could be made more sensitive to concerns of such groups to address their individual needs and misgivings.

The conscious mind and its aspects, such as choice and consent, are poorly defined, not universally agreed upon, and have broad practical implications on individuals and society. With advancing experimental designs, methodology, and scientific ideas, addressing the nature of consciousness and its influence on human health and social wellbeing becomes more important and more feasible. The philosophical debate surrounding consciousness and mind–body dualism has raged for millennia with radically differing views on the importance of a proposed immaterial mind and the material nature of the brain. Much of this debate depends on whether the physical structure and electrochemical constituents of the neurons and the brain they comprise are causally sufficient to predict future behavioral or motor output [5]. Any fundamental discussion of behavioral and motivational states or traits must consider the meaning of agency and the freedom of choice. In *Phaedo*, Plato argues that the soul is a separate, non-physical entity which is the seat of our thoughts [6], and René Descarte claimed in his *Meditations on First Philosophy* that the pineal gland of the brain was the location of a mysterious junction between the nonphysical soul and the physical body [7]. In the fourth century BCE, Aristotle pointed to the effect of hindrances that prohibit us from doing or choosing while leaving room for unconstrainted actions [8]. In the thirteenth century, Thomas Aquinas argued that humans have set goals which they seek, but how those goals are achieved is left to individual choices [9]. In 1739, David Hume outlined the compatibilist view of free will and determinism in “A Treatise of Human Nature”, suggesting that action without respect to cause is impossible and thus all actions are determined by the causes that precede them while simultaneously being the logical choice of a free mind [10]. A will that is completely free would be disconnected from and not responsive to the events leading to an action or following from that action, thus being stochastic and unpredictable. Such a freedom of will would be incapable of functioning in a physical, finite world of hunger, danger, and urges that drive our survival and reproduction. In this sense, a truly free will cannot exist. For this reason, the semantics of the will must be carefully considered. In practical life, our actions are constrained by the events leading up to those actions and are mere responses to those actions. Does this suggest that our agency as conscious individuals is limited or constrained? Can there be free agency in organisms with limited sensory abilities, limited capacity for accurate memory, and limited potential to act or affect their environment? While these questions have a philosophical root, their practical effects on the behaviors and policies of human society have broad implications on health, economics, and even justice. By looking at various factors—biological and environmental, categorical and circumstantial—that inform our sense of agency and limit our actions, scientific investigation of practical questions of the nature of free agency in both health and disorder can be pursued. In so doing, our understanding of the will as binary may dissolve, replaced by other representations such as a gradient of a more or less constricted agency, one governed by physiological, genetic, and environmental factors that may be altered through intentional, clinical intervention; or a binary quale may be confirmed. At the least, perhaps the problem will be understood in greater detail through such efforts at scientific pursuit.

## 2. Consciousness as Epiphenomenon

In the modern interpretation of neuroscientific data, our understanding of consciousness is described as an epiphenomenon related to the complexity of cortical information processing. In the simplified three steps of sensory input, cortical processing, and motor (or emotive) output, consciousness is how our brain observes the processing that occurs within the cortex. Consciousness, in this model, is not involved in any control over brain processes, and is instead an observer that has no impact on the stream of information in front of it. If we take the highly simplified analogy of the brain as a computer, where the activity of each neuron resembles a 1 or 0 corresponding to its action potential, then consciousness can be seen as the digital image that evolves from the aggregated and sequenced 1 s and 0 s that are occurring in our cortex; that is, a separate and holistic means of interpreting the flow of information. As an epiphenomenon, consciousness may be outside of the influence of survival and sexual fitness that predicate generational change, while some may argue for the implicit role of evolution in all aspects of behavior [11]. The role of consciousness as a mere consequence of short-term memory encoding has also been described and aligns with this epiphenomenal concept [12]. The fidelity of an organism’s consciousness in this model would be based on the complexity of the processing that occurs within the organism, creating a metaphorical image of greater or lesser detail based on the quantity of 1 s and 0 s available. If the quantity of information or the complexity of activity is a criterion for such an emergence, our reliance on a particular structure such as the brain as a singular seat of consciousness may be a mistake, because the fundamental processes and mechanisms of activity and information exchange can occur in innumerable systems [13]. This may result in a broad and inclusive definition of “consciousness” which may be present in systems, or even subsystems [14], ranging from physical- to field-based.

In such an epiphenomenal system, the philosophical concepts of both fate and choice can be simultaneously present in a semantic sense. Due to the uniqueness of each system, self-determination can occur due to the unique patterning of the systems present, creating a consciousness unique to the activity of that system in that moment in time. In the simple three steps of input, processing, and output, each individual system may possess structures or functions that vary in each of these steps, leading to a unique behavioral output which is our most prominent measurement of consciousness. However, all of the physical processes involved in these steps are still based on deterministic laws of particle interactions. In this way, an emergent consciousness may be considered to be compatible with both determinism and a choice that is specific to every separate point in space and time.

## 3. Constraints on Free Will in Health and Disorders

Regarding the nature of our mind–brain duality, neuroscientists have identified numerous dual natures of the brain, including conscious and unconscious, sympathetic and parasympathetic, left and right hemispheric lateralization of functions, cortical and subcortical, and so on. Some of these dualist functions can be attributed to scientists’ penchant for applying categorizations to information and data that exist organically across a spectrum, but these categories also point to true antagonistic balancing functions in a system based on tipping points that will determine ultimate output.

Cortical processes and autonomic functions are one such dual aspect that provide separate but interwoven functions of the brain, influencing each other but with very little direct interconnection. Cortical (often considered “conscious”) thought and autonomic responses can often limit each other through contradictory outputs, and a number of neuropsychiatric conditions—historically considered to be part of an ethereal “mind” or “spirit”—are now firmly placed within the realm of medicine and the biological sciences, with documented effects on these systems. Depression, anxiety, and post-traumatic stress disorder (PTSD) are a few such conditions that are impacted by these dual pathways of the brain. As the biological sciences continue to incorporate and define these as physical conditions, the question arises as to the potential for scientific investigation into the nature of the mind, free agency, the will, and determinism.

Two afferent (sensory) pathways exist in the human nervous system. These pathways target separate means of output, one through the autonomic functions that produce “unconscious” (i.e., subcortical) responses and the other which synapses on various cortical regions with associative connections that lead to intentional, cognitive, willed, or “conscious” action in response to environmental stimuli. Autonomic functions are only consciously realized after their output function has been facilitated through limbic pathways or hormonal output impacting visceral physiology, with effects that are later consciously sensed. While this can be interpreted as a “two brain” hypothesis of CNS output, there are also prolific, if not robust, interconnections between the cognitive and autonomic networks of the brain. Subsets of the human population are capable of voluntarily influencing autonomic tone such as vagal nerve activity and stress responses, including immune cell proliferation and activation states [15]. Such voluntary control over autonomic output has implications for a variety of neuropsychological disorders. Other examples point to the importance of genetics and neural developmental differences in homeostatic functions resulting in constrained capabilities [16,17,18]. Published studies on the electrical stimulation of regions of the brainstem that impact autonomic function support therapeutic effects for conditions such as depression [19], while other therapies attempt to alter brain connectivity over time using consciousness as an intervention for depression [20]. On a fundamental level, can the transfer of ideas alter individual volition, and how does this inform the discussion of free will, human health, and evolution [21]?

These scientific and therapeutic endeavors inform the debate on the nature of the conscious will, suggesting that our wills are constrained by environmental conditions, autonomic activity, and neural pathway connectivity resulting in a limited range of possible outputs. Reward-based learning mechanisms facilitated by dopamine activity and external factors can alter that range of outputs to change our conditioned responses to environmental stimuli. In this way, neuropsychological disorders can be considered to lead to a will that is further constrained by the environment and neural functions resulting in differences in motor and emotive output. These pathological states of the will and consciousness may result from dysfunctions or developmentally conditioned activity, genetic factors, environmental conditions, and cultural influence [22].

## 4. Consciousness as Fundamental

Alternative ideas of consciousness that are supported by clinical reports and rigorous analysis must be considered for a fully scientific evaluation of the topic. It may be possible that consciousness is not an epiphenomenon within the described three steps of input, processing, and output. Instead, in an analogy to aspects of the world of physics such as dark matter or dark energy and their relation to more familiar physical matter, consciousness may be a fundamental aspect of its own that interacts with the brain in an as-yet unclear manner, creating a materially or fundamentally “dualist” notion of the mind and body. In such an interpretation, a fundamental consciousness, whether it exists as a separate form or field, can separately interact with and influence any of these three steps. This influence could occur on a scale from subtle to profound, or not at all, leaving intact the prevalent deterministic view of particle interaction in the brain while allowing for free will. To continue the metaphor of the brain as a computer, the physical processes of the brain may continue to produce the digital 1 s and 0 s based on well-known physical laws, but consciousness may exist at a different metaphorical and occasionally overlapping “analog” signal, one which may occasionally influence the digital 1 s or 0 s to change. In this way, the metaphorical image produced by the consciousness is not an epiphenomenon or emergent property of digital 1 s and 0 s of the brain, but its own rich quale in parallel with brain processes and toward which our scientific instruments are not tuned, much like the dark matter of physics. In this interpretation, compatibilism is also intact, because the interaction of the metaphorically analog consciousness and the digital brain signals are not necessarily constant or mutually overriding. Additionally, there exists the potential for great value in identifying scientific methods for recording and investigating phenomena related to unexplainable interruptions or unexpected results in a simple three-step understanding of brain processes. Case series reports may be the first records of the intervention of this fundamental form of consciousness while providing important information to identify scientific means of falsifiable testing. Much like the difficulty in directly identifying dark matter other than through its gravitational effects, this interpretation would indicate that we are only observing the impact of a real, separate form of consciousness through its effects on easily observable physical systems such as behavior.

There is room, of course, for other iterations of the mind–brain phenomenon and the deterministic and/or free interpretation of decision making, and my intention is not to discount those without consideration [23]. Observations of functional brain anatomy [24] and electromagnetic field activity [25] reveal important and practical aspects of conscious processes and inform the discussion. Furthermore, this is to say nothing about the timing of consciousness relative to brain function, such as advances or delays in conscious awareness [26,27,28,29,30]. As examples, the prevailing statistical interpretation of quantum physics has been argued to allow for non-deterministic processes in brain activity and thus creating choice, while the coherent quantum movements of cellular structures have been viewed as providing a method for interactions with unrecorded fields of a separate, fundamental consciousness [31,32]. Careful experimental designs have led to interesting correlations, revealing advances in our ability to test such hypotheses [33,34,35], and showing direct implications for human health [20,36,37,38].

## 5. Practical Consequences of Our View of Consciousness

Regardless of the source of consciousness, it is clear that consciousness and decision-making play a critical role in human health. It can be argued that many neurological disorders, including psychiatric and behavioral disorders such as schizophrenia, can be interpreted as dysfunctions of the consciousness process. This raises questions regarding not just the qualities of consciousness, but the potential to describe quantities of consciousness. Humanity has long implied that there are levels of consciousness coinciding with a hubristic hierarchy of the evolution tree, with humanity placed unquestionably at the top. This description allows and facilitates interpretations of “the other” as less important within an ad hoc value system. This interpretation argues that non-human animals naturally have less consciousness than *Homo sapiens* and can lead to a conclusion that some humans have less consciousness as a result of differences in brain complexity or brain functionality, including those experiencing disease states. The corollary possibility is that, within this value system, we ultimately identify others that could possess “more” consciousness than we do. How would we react to a discovery that a known or yet unknown species might possess quantifiable and objective levels of consciousness equal to or beyond our own [39,40,41]?

Alternatively, some may argue that this hierarchical value system does not represent the true nature of consciousness. Perhaps all objects possess the same quale of consciousness and only vary in their ability to outwardly represent that consciousness as observed and interpreted by humans. A fundamental and intrinsic quale attributable to consciousness may be the case when considering the complexity of system activity at different scales or based on the potential of a separate field theory of consciousness. As we view systems in greater detail, complexity tends to increase and often in an exponential manner. In this way, as we investigate systems to smaller scales, it may be that there is no appreciable difference in epiphenomena that emerge from such complexity. Or, as we view systems as an integrated whole from a broad perspective, complexity may similarly scale, leading to an interpretation of consciousness beyond the arbitrary boundary definition of a single organism. If consciousness is a result of complexity and interconnected interaction, what systems at different scales may qualify as conscious? If consciousness is a separate field or particle that interacts in a manner we do not yet understand, it is possible that this field is not confined to traditional biological boundaries. We often study behavior as a correlate to studies of consciousness. However, it may be that trees and rocks interact with or possess such a consciousness field but these things do not outwardly convey our expected signs of consciousness due to limitations on their physical structures and their ability to output behavior on timescales to which human consciousness is accustomed [42,43]. New frontiers in computation and computational neuroscience are raising questions about the ability of software algorithms operating on electronic transistors to achieve popular notions of consciousness, and these require careful consideration as we seek definitions for and implications of consciousness [44,45,46]. The models produced by computational neuroscience can also have an impact on our understanding of system complexity and emergent properties.

## 6. Summary

As science adopts investigations of free agency and consciousness, the practical questions and moral implications of the assignment of responsibility for actions arises based on the ability of an individual to choose one course of action over another instead of simple determinations based on the needs of the many [47]. By describing pathways of the central nervous system—including automatic, autonomic, and cortical networks—and their resulting motor and emotive outputs, researchers have the potential to define the impact of disease states and dysfunctions on an individual’s free agency. In so doing, the potential exists to clarify the source of individual agency and social responsibilities to individuals and the group while defining pathological variations in consciousness and volition resulting in salient and practical implications for human health and wellbeing. On a philosophical level, by defining the neurological ability to identify input signals and coordinate “appropriate” responses, does science have the ability to validate Platonic ideals and Kierkegaardian categorical imperatives, or will it discover Aristotelian volition and Nietzschean relative moralism?

Such questions are not sophistry and should not be restricted to the philosophy classroom, because these differences in interpretation influence our social interactions, healthcare and bed-side approach, policy, and political rhetoric among many pivotal aspects of human life. As such, human health and wellbeing are directly impacted by arguments surrounding the nature of consciousness, as are the systems that surround and support individuals and society at large.

In this Topical Collection, I intend to provide a peer-reviewed space for practical and hypothesis-driven input and discussion on the sources of decision-making of the human individual and other organisms or systems that might inform our interpretation of activities related to consciousness, the mind and brain, and beyond. This Topical Collection is also intended to emphasize the impact that the discussion of consciousness and decision-making has on human health and social interaction and describe the importance of educational efforts for clinicians and the public in what has traditionally been considered a purely philosophical, or even frivolous, topic.
